# 

**Published:** 1895-03-09

**Authors:** 


**Notices of Births, Deaths, and Marriages** are inserted in this column at a charge of 2s. 6d. for an announcement not exceeding four lines.

**Notice to Advertisers and Subscribers.**

All Advertisements, Orders for Copies of The Hospital and Business Communications should be addressed to **The Manager, NOT TO THE EDITOR,** The Hospital, 428, Strand, London, W.C.

The Cost of Subscription, post free, is as follows:—Per week, 2½d.; per quarter, 2s. 8d.; per half-year, 5s. 3d.; per annum, 10s. 6d. Foreign:—Per Annum, 15s. 2d.; payable, in all cases, in advance.

**NOTICE TO CORRESPONDENTS.**

All MS., Letters, Books for Review, and other matters intended for the Editor should be addressed The Editor, The Lodge, Porchester Square, London, W.

The Editor cannot undertake to return rejected MS., even when accompanied by stamped directed envelope.

**"Burdett's Hospital Annual," 1895,**

Ready Early in March.

Sixth year of publication. About 1000 pp. Scarlet cloth, gilt lettered. Price 5s. A most complete guide to British, Colonial, Indian, and American Hospitals, Dispensaries, Nursing and Convalescent Institutions, and Asylums. A few copies of the "Annual" for 1894 may still be obtained on application to the Publishers, The Scientific Press (Limited), 428, Strand, London, W.C.

**NOTICE.—The Index for Vol. XVI. (ending September 24th, 1894) is on sale at the Office of this Journal, Price 2½d., post free.**

### SCRAPS AND GLEANINGS.

The Paris Academy of Medicine has received £20 from Baron Larrey to found a prize for medical statistics.

The boys of the Dover College have given a cot to the local hospital, and have also arranged for its endowment, for the estimated cost of which for the past year their contribution of £25 has proved a welcome one.

Two benefactors to the Mount Pottinger Hospital for Women and Children have made testamentary arrangements by which the hospital will benefit to a considerable extent—Mr. A. T. Macaulay and Mr. Thomas Holden—the former of whom generously bequeathed £1,000.

Towards the new ward and the other improvements at the Sick Children's Hospital, Glasgow, special donations have been received to the amount of £2,240. The expenses incurred by the extensive sanitary alterations have to be met out of the ordinary funds of the institution.

The National Training School of Cookery has just inaugurated classes for the instruction of volunteers in all branches of cookery. It is announced that any volunteer desirous of attending these classes should send his name to the orderly-room through the officer commanding his company.

The returns of the medical staff of the Ulster Hospital for Women and Children show that the extern department continues to increase with great rapidity, 8,628 cases receiving treatment during the last 12 months, a fact which speaks for itself as to the extent to which the public are utilising this department of the hospital.

At the annual meeting of the governors of the Rotherham Hospital and Dispensary a suggestion was made that the workmen's representatives might assemble together and consider the best means to increase their subscriptions. About 150 workmen acted upon this suggestion, and a very unanimous and earnest desire was manifested to help those who had charge of the institution to rid themselves of the burden of an existing debt of £900.

### The Student of Medicine.

### APPOINTMENTS.

A. Byers, M.B., Ch.B.Vict., B.Sc.Lond., appointed Junior Resident Medical Officer to the General Hospital for Sick Children, Pendlebury, Manchester. Mr. T. V. Cunliffe, appointed House Surgeon to the Manchester Royal Infirmary. Mr. J. S. Dockeray, appointed House Surgeon to the Manchester Royal Infirmary. Mr. J. C. Edgar, appointed Clinical Assistant to the Convalescent Hospital of the Manchester Royal Infirmary. Mr. T. B. Flint, appointed House Physician to the Manchester Royal Infirmary. Dr. Grant, appointed House Surgeon to the Glasgow Maternity Hospital. Dr. Hamilton, appointed Medical Officer for the Workhouse of the Rathdown Union. J. S. Hinnell, B.A.Cantab., M.D., M.R.C.S.Eng., appointed Assistant Medical Officer to the Suffolk General Hospital. Mr. R. T. Hughes, appointed House Surgeon to the Manchester Royal Infirmary. A. R. Iles, M.R.C.S.Eng., L.R.C.P.Edin., appointed Honorary Surgeon to the Taunton and Somerset Hospital. H. W. G. Lander, M.B., C.M.Edin., appointed Assistant House Surgeon to the District Hospital, West Bromwich. Dr. Macdonald, appointed Medical Officer for the Parish of Kiltarlity. P. M'Dougall, B Sc.Lond., M.B., Ch.B.Vict., appointed House Surgeon to the Manchester Royal Infirmary. C. H. Melland, M.R.C.S., L.R.C.P.Lond., appointed House Physician to the Manchester Royal Infirmary. G. G. Morrice, M.A., M D.Cantab., M.R.C.P., appointed Physician to the Salisbury Infirmary. W. G. Parkinson, M.R.C.S., L.R.C.P., appointed House Surgeon to the Hospital for Women and Children at Leeds. Z. Prentice, M.R.C.S.Eng., L.S.A., appointed Surgeon to the Kent and Canterbury Hospital. H. W. Smart, L.R C.S.I., L.M., appointed Medical Officer for Newry No. 1 Dispensary District. S. T. Taylor, M.B.Lond., L.R.C.P.Lond., M.R.C.S.Eng., appointed Medical Officer of Health to the Chingford Urban District Council. A. G. Young, M.B.Glasg., appointed Medical Officer and Public Vaccinator for the Rillington District of the Norton Union.

St. Thomas's Hospital.—The following gentlemen have been selected as House Officers from Tuesday, March 5th, 1895:—Resident House Physicians: E. A. Saunders, M.A., M.B.,

THE STUDENT OF MEDICINE—*continued.*

B.Ch.Oxon., L.R.C.P., M.R.C.S. (extension), and G. G. Genge, L.R.C.P., M.R.C.S. (extension). Non-Resident House Physicians: F. J. Brakenridge, L.R.C.P., M.R.C.S., and J. W. Laver, L.R.C.P., M.R.C.S. House Surgeons: G. J. Arnold, L.R.C.P., M.R.C.S., R. Fox Symons, L.R.C.P., M.R.C.S., A. E. Russell, M.B.Lond., L.R.C.P., M.R.C.S., and H. W. Harding, L.R.C.P., M.R.C.S. Assistant House Surgeons: E. O. Thurston, L.R.C.P., M.R.C.S., and A. L. Home, L.R.C.P., M.R.C.S. Obstetric House Physicians: Senior, W. E. F. Tinley, M.B., B.S.Dur., L.R.C.P. M.R.C.S.; Junior, S. W. F. Richardson, M.B., B.S., B.Sc.Lond., L.R.C.P., M.R.C.S. Clinical Assistants in the Special Department for Diseases of the Throat: T. O. Halliwell, L.R.C.P., M.R.C.S. (extension), and J. L. Prain, L.R.C.P., M.R.C.S. Clinical Assistants in the Special Departments for Diseases of the Skin: H. H. Haward, B.A.Cantab., L.R.C.P., M.R.C.S. (extension), and L. A. R. Wallace, B.A., M.B., B.Ch.Oxon., L.R.C.P., M.R.C.S. Clinical Assistants in the Special Departments for Diseases of the Ear: F. V. Milward, B.A., M.B., B.C.Cantab., L.R.C.P., M.R.C.S., and H. J. Davis, L.R.C.P., M.R.C.S., B.A.Cantab. Clinical Assistant in the Electrical Department: A. Barry Blacker, M.D., B.S.Dur., L.R.C.P., M.R.C.S. (extension).

### VACANCIES.

### Resident Medical Officers.

City of London Hospital for Diseases of the Chest, Victoria Park, E.—House Physician. Appointment for six months. Board, residence, and allowance for washing. Application to the Secretary at the Office, 24, Finsbury Circus, E.C., by March 15th.

Northumberland County Lunatic Asylum, Morpeth.—Assistant Medical Officer; unmarried. Salary £120 per annum, increasing £10 yearly to £150, with furnished apartments, board, and lodging. Applications (marked outside "Medical") to Dr. McDowell at the Asylum by March 11th.

Royal United Hospital, Bath.—House Surgeon, must be M.R.C.S.Eng. Salary, £60 per annum, with board, lodging, and washing. Appointment for six months. Applications to W. Stockwell, Secretary-Superintendent, by March 20th.

Sussex County Hospital, Brighton.—Fourth Resident Medical Officer, doubly qualified, unmarried. Emoluments are board, residence, and washing, and a salary not exceeding £30 per annum. Applications to the Secretary by March 18th.

Victoria Hospital for Consumption and Diseases of the Chest, Edinburgh.—Resident Physician. Appointment for six months. Apartments, board, and washing, with allowance of £2 monthly for conveyance in connection with outdoor department. Applications to the Honorary Secretaries. Messrs. Wallace and Guthrie, 1, North Charlotte Street, Edinburgh, by March 11th.

### Non-Resident Medical Officers.

Cape Colony.—Medical Officer of Health for Cape Colony. Salary £1,000 per annum. Applications to the Agent-General, 112, Victoria Street, S.W., or the Colonial Under-Secretary, Cape Town, by March 15th.

Hospital for Sick Children, Great Ormond Street, Bloomsbury, W.C.—Medical Registrar and Pathologist. Appointment for one year. Honorarium, 50 guineas. Applications to the Secretary by March 26th.

Wimborne Minster Urban District Council.—Medical Officer of Health. Salary, £30 per annum. Applications to Herbert William Dibben, Clerk, Urban District Council Offices, Wimborne Minster, by March 11th.

GREAT ORMOND STREET, Bloomsbury, W.C.

The periodical VACANCY for RESIDENT MEDICAL OFFICER will occur on April 10th next, the appointment being for six months, with salary at the rate of £100 per annum, and Board and Lodging.

Candidates must be legally qualified, and be, or become, members of the British Homœopathic Society.

Applications for the post, with 16 copies of testimonia's, must be sent in to the Secretary-Superintendent by March 14th.

G. A. CROSS,

Secretary-Superintendent.

(1,183)

### THE HOSPITAL FOR SICK CHILDREN,
GREAT ORMOND STREET, Bloomsbury, W.C.

